# 

**Published:** 1895-06

**Authors:** 


					﻿Vol XV.
JUNE, 1895.
NO.-6."
THE
pOMDEDPATHlC pHY^lClAfJ,
A Monthly Journal of Homoeopathic Materia Medica
and Clinical Medicine.
"Be is a freeman whom truth makes free, and all are slaves beside."
"Seek the Truth: come whence it may, cost what it will."
EDITED JWD PUBLISHED BY
WALTER Kf. JAMES, M.; IX
PHILADELPHIA :
ISTo. 1231 LOCUST STREET.
•	.LONDON:
ALFRED HEATH A CO., Sole Agents tor Obeat Bbitaim,
114 Ebury Street, Eaton Square.
JK^-Yearly Subscription, $2.SO, invariably in advance. Single numbers, 2S etc.
Entered al tbe Philadelphia Paaa-Otte* M Bianl-CUaa Mall Matter.
				

